# How should risk be communicated to children: a cross-sectional study comparing different formats of probability information

**DOI:** 10.1186/1472-6947-9-26

**Published:** 2009-06-05

**Authors:** Fiona Ulph, Ellen Townsend, Cris Glazebrook

**Affiliations:** 1 School of Psychological Sciences, University of Manchester, Oxford Road, Manchester, M13 9PL, UK; 2 School of Psychology, University of Nottingham, Nottingham, UK; 3 Division of Psychiatry, University of Nottingham, Nottingham UK

## Abstract

**Background:**

Newborn screening, which identifies inherited disorders and sometimes carrier status, will increasingly involve health professionals in the provision of appropriate information and support to children and their families. The ability to understand carrier results relies on an understanding of probabilistic terms. However, little is known about how best to convey probabilistic medical information to children. Research with adult populations suggests information format significantly affects comprehension. This study aimed to explore which presentation format is most effective in conveying probabilistic information to children.

**Methods:**

A probabilistic task based on the cup game was used to measure which of five different formats was associated with greatest understanding in children aged 7–11 years old (n = 106). Formats used were verbal labels (e.g. rarely, sometimes), percentages, proportion-word (e.g. 1 in X), proportion-notation (e.g. 1:X) and pie charts. There was also an additional mixed format condition. In each trial a picture was presented of three cups, each with a different probability depicted beneath it, and the child was asked to select which cup was most likely to contain the ball. Three trials were presented per format. Children also rated how certain they were that they had answered correctly.

**Results:**

There was a significant relationship between format and comprehension scores. Post hoc tests showed children performed significantly better when probability was presented as a pie chart, in comparison to percentages, proportion – notation, proportion-word and mixed format trials. Furthermore, most children (84%) got all trials correct for this format and children were significantly more certain that their response was correct in the pie chart trials compared to all the other formats (p < 0.001). Significant positive correlations were found between self-ratings of certainty and comprehension of verbal labels, percentages and pie charts. Older age was also associated with better performance on all formats except percentages. Overall comprehension was calculated by summing the scores for the individual trials and this was independently associated with older age and higher IQ.

**Conclusion:**

The results suggest that 7–11 year olds can understand probability information, but that the format used will significantly affect the accuracy and confidence with which children in this age group make judgements about the likelihood of an event. Of the formats studied, pie charts appear to be the optimal method of presenting probabilistic information to children in this age group. Health professionals and designers of health messages should be cognisant of this when communicating medical information to children aged 7–11 years old.

## Background

Historically, children (people below the age of 16) have been regarded as passive recipients of health care, with information, decisions and consultations directed towards their parents [1]. The Gillick Case in the UK [2], in which the right for a child below the age of 16 to be prescribed contraception without parental knowledge was recognised, challenged this. In doing so it set the precedent that children who could demonstrate Gillick competence should be involved in health care decisions. Subsequently, a number of policies were issued both within the UK and internationally which stipulated that children should, as far as developmentally possible, be encouraged and supported to take an active role in their health care [3,4]. More recently, the General Medical Council has advocated the inclusion of *competent *children in their own health care decisions [5].

Gillick competence has been adopted into law within the UK, Australia and Canada and is based on proving that the child is rational and able to communicate a decision which evidences that they have considered the consequences of such a choice and any alternative options [6,7]. Thus, a child's competence is determined by their ability to understand the implications of their decision. How effectively the information regarding the risks and likely outcomes of different decisions has been communicated to children will arguably significantly influence their ability to make informed decisions regarding their health care [8]. Such a belief underpins the research interest in how best to communicate health information to children. An omission within this field is an examination of how best to present probability-based information to children.

Risk comprehension is central to many decisions about health [9] and is key to informed consent to treatment [10,11]. Research regarding adults' understanding of risk messages suggests the way risk information is conveyed impacts on an individual's ability to appraise different treatment regimens [12-14], change behaviours [15,16], and choose treatment outcomes [17]. This has lead to a proliferation of research examining how best to convey health risk information to adults [18-23]. Verbal labels [24,25], proportions [26,27], percentages [28], graphical representations [16,29,30] and a mixture of formats [31,32] have all been proposed as optimal formats. What is not clear from the existing research is the most effective way in which to convey probabilistic messages to children and whether they will similarly be affected by the format used.

Studies based on Piaget's theory of cognitive development suggests that children cannot understand probability until approximately 11 years of age [33] and the UK mathematics curriculum largely reflects this Piagetian staging [34,35]. Although Piagetian theory provides a framework to explore the development of probability comprehension [36] an analysis of the evidence and subsequent research indicates this theory may be too simplistic [37]. Indeed, neo-Piagetian research indicated that, with appropriate guidance, children as young as 7 years old can understand probability [36,38,39]. Research into probability judgements is now characterised by a focus on the idea that intuitive processes are often used to solve probabilistic tasks and variance in performance is to be attributed to the individuals' ability to select the relevant information from competing stimuli [36]. This work indicates that the ability to solve probabilistic tasks is present early in development and that children also appear to be vulnerable to task biases that affect adults. Thus, it is likely that the ability of children to understand medically related probabilistic information will be influenced by a number of factors, including the presentation of the task.

More recently, a study which compared the ability of children with adults when making expected value judgements concluded that children as young as five or six can understand probability [40]. Children were found to employ similar strategies to adults and also understood that probability was an abstract concept. A cross-sectional survey study of 10–15 year olds and adults found large variation in the percentages attributed to verbal expressions, especially in the 10–15 year olds' responses [28]. Participants had most difficultly when distinguishing between the verbal labels "possibly" and "probably".

The adult literature indicates that visual representations are beneficial for conveying probability to those with low literacy and numeracy skills [29,41] and for retaining attention [41]. The latter finding has been explained in terms of the information being presented in concrete terms. This may be particularly crucial when communicating with children, particularly if formats which are familiar from school lessons are used (e.g. pie charts).

Although it has been acknowledged that risk communication is central to conveying medical information and patient decisions, the context for the present study was the introduction of newborn screening utilising genetic testing. Effective communication of probability is seen as central in genetics [42-44]. The implementation of newborn genetic screening programmes, which identify inherited disorders and sometimes carrier status in newborns, will result in a generation of children about whom genetic information is known prior to an age at which the individual concerned could understand the implications of being a carrier.

The ability to appreciate the random nature of inheritance and the reproductive implications of carrier status is reliant on probabilistic understanding. For example, a carrier of a gene for an autosomal recessive disease such as cystic fibrosis or sickle cell, (diseases screened for currently in the UK newborn screening programme), has a one in four chance in each pregnancy that their child will be affected with the disease if their partner is also a carrier. Additionally, there are the relative likelihoods of their partner being a carrier as a result of the prevalence in the community, or of their wider family members being carriers as the gene has been identified within the family. Thus, it is likely that any communication of carrier status to these children will necessitate an explanation of probability [45].

Although some parents will feel confident in conveying information about carrier status to their child, evidence suggests the communication of genetic information within families is limited [44,46] and problematic [47]. Many parents report that they find it difficult to inform children of their results and to be frank with them about the effects of the illness [47,48]. Thus, it is likely that health professionals will be called on to support parents with information giving [49]. Recent research suggests that children aged 7–11 years old are in the process of constructing knowledge about genetics and it is at these ages that children could benefit from guidance (Ulph, Glazebrook & Townsend, Children's understanding of genetic concepts, submitted). What is uncertain is whether or how children could understand the probabilistic nature of genetic information and the most effective way to convey this information. The aim of this study was, therefore, to determine whether children aged 7–11 are able to make probabilistic judgements and whether the format of the presentation of probabilistic information affects this ability.

## Methods

### Participants

One hundred and six children aged 7 to 11 years were recruited from 11 schools in the East Midlands area of the UK. Schools were sampled to represent a range of academic achievements, proportions of children receiving free school meals, and percentages of ethnic minority children enrolled (table 1). The percentage of children receiving free schools meals is routinely used by researchers as a measure of socio-demographic status [50]. Academic standards are indicated by the percentage of children achieving level four at key stage two. Within the UK children are tested at key stages throughout their education. Performance within these key stages is often used as a measure of whether pupils within schools are reaching designated levels and, therefore, reflects the academic achievement of pupils within the school. Children aged 7–11 years old are within key stage two. Level four at key stage two represents a level that is expected of each eleven year old.

**Table 1:** Information about the schools included in sample.

School Code	No. pupils in sample	Date of school report	% free school meals (20%)	% ethnic minority	**Percentage of pupils achieving level 4 at key stage 2**.		
					
					English (75%)	Maths (72%)	Science (85%)
**01**	17	2005	10	3	85	74	85

02*	4	N.A.	Below Average	Not avail	N.A.	N.A.	N.A.

**03**	3	2001	59	74	71	94	94

**04**	18	2005	15	2	70	87	95

**05**	7	2001	7	10	91	87	94

**06**	16	2003	18	8	71	69	93

**07**	2	2002	33	9	65	63	77

**08**	8	2003	21	3	83	82	97

**09**	16	2001	14	1	59	65	57

**10**	10	2003	18	67	50	53	82

**11**	7	2003	Above average	10	65	56	67

* No key stage two results are reported for this school as it is an infant school. The information regarding free meal and ethnic origin percentages was provided by the head teacher, thus there is no report date provided

Parents of children who were in the appropriate age-range and attending one of the study schools were sent study information at least one week prior to the researcher (FU) attending the school. Children whose parents agreed to their participation were seen individually in a setting chosen by the school. Research suggests that children need clear explanations about why they are being asked to participate in research and what role they should take [51]. Therefore children were given verbal and written information about the study, and shown the measures with simple explanations of each, before being asked to assent to participation. After assenting, the child completed the tasks in the order they chose. The age, gender and BPVS scores for the participants are reported below (see table 2).

**Table 2:** Demographic information of participants.

	Age 7–9 (N = 32)	Age 10–11 (N = 74)	Total
**Gender**			
**Male**	18 (17%)	33 (31%)	51
**Female**	14 (13%)	41 (39%)	55

**Mean BPVS score (SD)**	100 (10.73)	101 (11.68)	101 (11.36)

**Median BPVS Score (range)**	100 (73–117)	100 (81–130)	100 (73–130)

**Median total score**	9 (5–17)	15 (5–18)	14 (5–18)

The median scores and ranges indicate this sample is representative of the population and includes sufficient variation to capture different abilities.

Participants were divided into two groups 7–9 and 10–11 years old based on the Piagetian theoretical assumption that within the ages 7–11 understanding would be most different between these two age groupings [33]. Although all ages of children were important to the study outcomes, more children were recruited into the older age group as probability understanding was hypothesised to be most developed in this group.

### Measures

#### Probability Task

**Figure 1:**
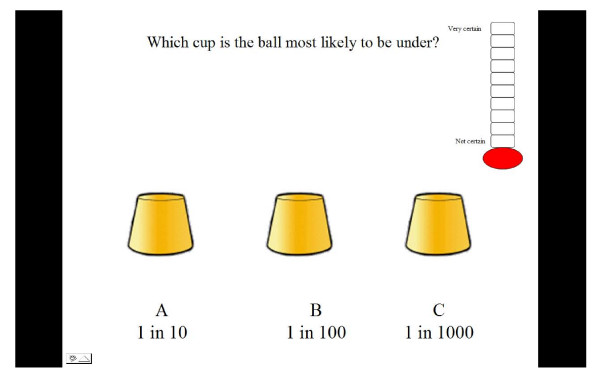
**Illustration of one cup game trial**.

**Figure 2:**
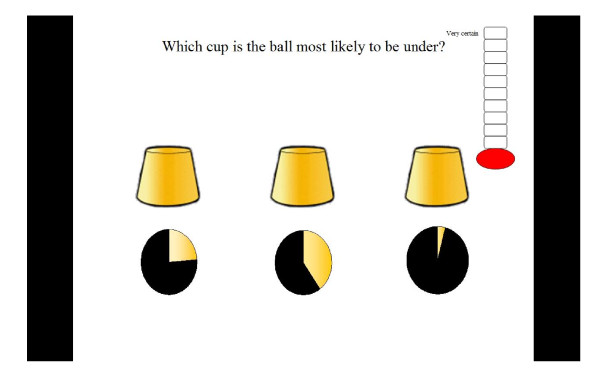
**Illustration of a pie chart format trial in which the light section indicates the likelihood of the ball being under that cup**.

A probability task was constructed which consisted of 18 trials. The task was designed to emulate a game many children may have witnessed called the cup game. The child was presented with a work book with pictures of three cups on each page [See Figure 1 and Figure 2].

**Figure 3:**
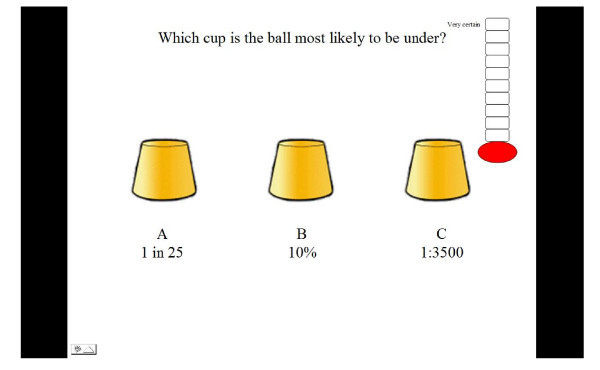
**Illustration of mixed format trial**.

Five different probability formats were selected based on a review of relevant literature which suggested these were regularly used in health information messages. These were verbal labels (e.g. rare, highly likely); percentages: pie charts and proportions depicted either as words (e.g. 1 in 10) or notation (e.g. 1:100). The verbal labels were not aligned with specific numerical information as, due to the large differences found in the numerical likelihoods attributed to verbal labels [51-55], it was believed that this could not be done in a meaningful way. Each trial within the five formats contained three different probabilities for children to compare. These were selected by a panel of experts in risk communication, developmental psychology and genetic communication. Probabilities were selected to test children's ability to understand probability terms presented in different formats. Thus some trials contained large differences (e.g. 33%, 57%, 18%) whilst in others the differences were small (e.g. 83%, 79%, 84%). Other trials were designed to examine whether children were fallible to common misperceptions such as 1 in 1000 being more likely than 1 in 10 (e.g. 1in10, 1in100, 1in1000) [56]. There were three trials for each format and three additional trials which contained a mixture of formats as illustrated below [See Figure 3].

The slides also contained a ten point certainty scale depicted as a thermometer. The inclusion of this scale is in keeping with previous probability research [37] and provided an indication of how comfortable the child was working with each format.

#### The British picture vocabulary scale (BPVS) (second edition)[57]

Previous research has illustrated the importance of measuring a child's ability to comprehend verbal task instructions and produce responses when examining children's knowledge [58]. The BPVS is a valid and reliable measure of receptive vocabulary in children and scores equate closely to verbal IQ. Total scores are age standardized to give IQ scores with an expected population mean of 100 (SD 15).

### Data collection

The tasks took approximately 20 minutes to complete. In each trial the child was asked to select the cup which was most likely to have a ball underneath it based on the probability provided under each cup. The children were asked if they recognised each format and whether they required an explanation. A uniform explanation was given if requested and this was recorded on their score sheet. For example, if children indicated they wanted an explanation of percentages they were told that it meant 'how often something would happen out of one hundred'. If the child selected the cup with the highest probability depicted below it the child was given one point. The order in which formats were presented was counterbalanced to minimise the effect of previous formats on performance. The total score a child could achieve for each format was 3 with a maximum total task score of 18. Next the child was asked to indicate how certain they were that their answer was correct by choosing a level on the certainty scale with higher levels reflecting more certainty.

To administer the BPVS, participants are read a series of words. After each word the participant is asked to select the picture which represents the word from a page containing four line drawings. Participants are not required to read or write and responses can be verbal or by gesture.

Children were encouraged throughout the task, but no feedback regarding the accuracy of their answers was provided. Following completion of the tasks, children were asked whether they had any questions about the research or the tasks that they had just completed. After answering their questions they were thanked for their participation.

### Analysis

Data were analysed using SPSS version 11.6. Histograms and significant Kolmogorov-Smirnov test results indicated that the data were not normally distributed. Thus, non-parametric tests were used to analyse the data. Friedman's tests were performed to analyse differences in performance and certainty across formats. Significant differences between formats were identified using post-hoc Wilcoxon signed rank tests applying a p value of 0.003 (Bonferroni correction). The Mann-Whitney U test was used to establish whether performance was affected by verbal ability, age group or the provision of an explanation. Effect sizes for both Mann-Whitney U tests and Wilcoxon signed rank tests were calculated by converting z scores into effect size estimates [59]. A hierarchical multiple regression enabled the assessment of the influences of age, verbal ability and gender.

### Ethics

This study was approved by the University of Nottingham's School of Psychology Ethics Committee.

## Results

There were no differences in BPVS scores or gender between the two age groups. Results of a Kruskal-Wallis test showed that the presentation order of formats did not affect performance so order of presentation was not included in analyses. A Mann Whitney U, performed to analyse the effect of high (≥ 100) or low (≤ 99) BPVS scores [60], found no significant effects of verbal ability on performance scores for any of the formats.

### Explanation of Formats

Uniform verbal explanations of formats were provided when requested. Table 3 reports the number of times explanations were given for each format.

**Table 3:** Frequency of explanation required per format for entire sample (n = 106)*.

	Frequency of explanation (%)	Relationship to performance
**Verbal label**	48	U = 1346.00, *p *= .67, *r *= -0.04

**Percentage**	6	U = 234.50, *p *= .28, *r *= -0.10

**Proportion-word**	27	U = 1017.50, *p *= .46, *r *= -0.07

**Proportion-notation**	36	U = 1133.00, *p *= .26, *r *= -0.11

**Pie chart**	16	U = 735.50, *p *= .78, *r *= -0.03

* With the exception of percentage in which N = 105

Percentage and pie chart formats required explanations least frequently, whilst label and proportions required the most. Two tailed Mann-Whitney analyses found no difference in scores for trials where an explanation had been given compared to those without explanation (see table 3 above).

### Format Effects

**Figure 4:**
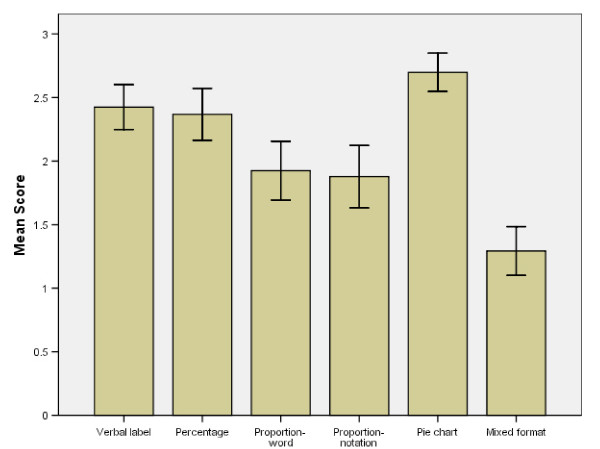
**Mean cup game score and 95% confidence intervals per format for entire sample**.

For each trial children could score 1 point for selecting the correct cup, creating a maximum score of 3 per format. The mean correct scores per format are presented in Figure 4 with error bars representing 95% confidence intervals. The graph illustrates that children achieved the highest scores with pie charts, followed by verbal labels and percentages.

The Friedman test showed a highly significant effect of format on comprehension (χ^2 ^= 126.61, df = 5, p < .001). Scores on the pie chart format were significantly higher than the following formats: percentage (z = 3.49, p < 0.001), proportion-word (z = 5.44, p < 0.001), proportion-notation (z = -5.11, p < 0.001), and mixed formats (z = -7.46, p < 0.001). Comparison between pie chart format and verbal labels failed to reach significance (z = -.63, p = .53). Children scored significantly worse when presented with mixed formats compared to verbal labels (z = -6.48, p < 0.001), percentages (z = -6.09, p < 0.001), proportion-word (z = -4.93, p < 0.001), and proportion-notation (z = -4.21, p < 0.001). Children found it significantly easier to choose the highest probability when data were presented as verbal labels compared to proportion-notation (z = -3.59, p < 0.001) and proportion-word (z = -3.48, p < 0.001). Performance was also better on percentages compared to proportion-notation (z = -3.10, p = .002). The direction of difference, significance values and effect sizes can be seen in table 4.

**Table 4:** Comparison of mean scores per format for entire sample on scale of 0–3 (difference, significance value, effect size).

Variable compared	Percentage	Proportion-word	Proportion notation	Pie chart	Mix
**Mean (SD)**	2.37 (0.92)	1.92 (1.20)	1.88 (1.28)	2.70 (0.78)	1.29 (1.00)

**Verbal label**	>	>	>	<	>
	*p *= .530	*p *= .001*	*p *< .001*	*p *= .005	*p *< .001*
	*r *= .06	*r *= .34	*r *= .35	*r *= -.27	*r *= .63

**Percentage**		>	>	>	>
		*p *= .005	*p *= .002*	*p *< 001*	*p *< .001*
		*r *= .27	*r *= .30	*r *= -.34	*r *= .59

**Proportion – word**			>	<	>
			*p *= .610	*p *< .001*	*p *< .001*
			*r *= .05	*r *= .53	*r *= .48

**Proportion – notation**				<	>
				*p *< .001*	*p *< .001*
				*r *= -.50	*r *= .41

**Pie chart**					>
					*p *= .001*
					*r *= .72

* Significant at *p *=< .003 level (Bonferroni correction)

Although children's performance in the mixed formats trials was significantly worse than other trials, this was not solely responsible for the finding that format affected comprehension. When these trials were omitted from the analysis the Friedman's test result was still significant (χ^2 ^= 53.55, df = 4, p < .001). The number of children who achieved maximum scores on each format these can be seen in table 5

**Table 5:** Number and percentage of children achieving maximum scores on each format with 95% confidence intervals.

	N	%	95% CI
**Verbal label**	69	65	55.92–74.08

**Percentage**	74	70	61.28–78.72

**Proportion-word**	48	45	35.53–54.47

**Proportion-notation**	53	50	40.48–59.52

**Pie chart**	89	84	77.02–90.98

**Mixed formats**	16	15	8.2–21.8

### Age and Gender Effects on Format

**Figure 5:**
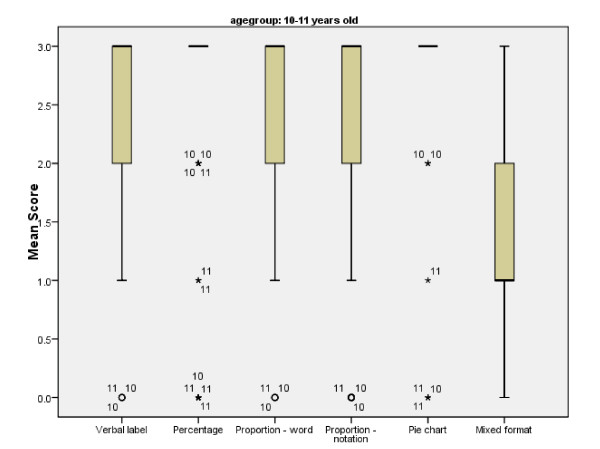
**Mean, range and interquartile range of cup game scores for children aged 10–11 years old per format**.

**Figure 6:**
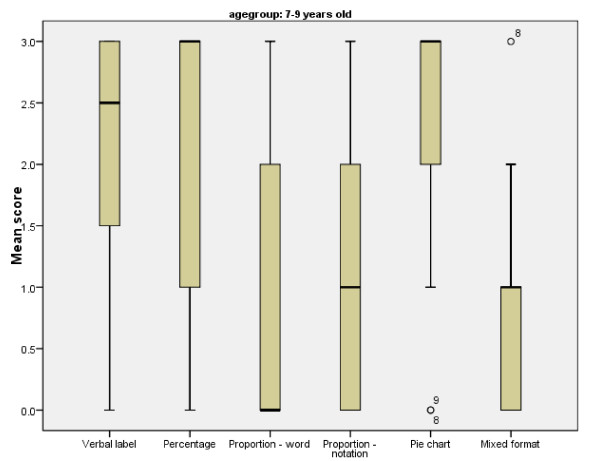
**Mean, range and interquartile range of cup game scores for children aged 7–9 years old per format**.

The older age group performed significantly better on all formats except percentages (U = 9 82.000, two tailed p = .086, r = -0.17) (Figure 5). An examination of the median and interquartile ranges in the graphs below highlights the variation in performance amongst the 7–9 year olds (see Figure 6).

The graphs also show that children in both age groups performed best on the percentage and pie chart formats, with least variation in performance in the pie chart condition. There were no effects of gender.

### Self-ratings of Certainty

For each trial presented, children rated how certain they were that their answer was correct on a scale 1–10. Children could score a maximum of 30 per format. These analyses were conducted on 101 children. Scores for five children were omitted from the analyses as they consistently selected maximum scores on all trials, showing no evidence of utilizing the measure to reflect their feelings of certainty.

A Friedman's test indicated a significant effect of format on certainty score (χ^2 ^= 125.81, df = 5, p =< .001). Table 6 displays the median certainty scores and interquartile ranges per format by age group. These data illustrate that children of both ages felt most confident that their answer was correct when viewing pie charts.

**Table 6:** Median and interquartile ranges of certainty scores per format by age group.

	7–9 years old (N = 29)		10–11 years old (N = 72)	
	
	Median	Interquartile range	Median	Interquartile range
**Verbal label**	21	15.5–24	21	17–24

**Percentage**	22	17.5–26	23.5	18.25–27

**Proportion-word**	22	17.5–27	19	14.25–24

**Proportion-notation**	20	15–27	18.5	15–23

**Pie chart**	26	24.5–30	24.5	18–28

**Mixed**	22	17–26	17.5	14–22

The significance of these differences was explored through Wilcoxon signed rank tests using a p value of 0.003 (Bonferroni correction). Children reported feeling significantly more certain of their answers when probability information was presented as pie charts compared to verbal labels (z = -5.72, p < .001, r = -0.57), percentages (z = -3.31, p < .001, r = -0.33), proportion-word (z = -7.04, p < .001, r = - 0.70), proportion-notation (*z *= -7.17, *p *< .001, *r *= - 0.71), and mixed formats (*z *= - 7.47, p < .001, r = -0.74). Children also felt more certain when making comparisons across percentages compared to verbal labels (z = -4.11, p < .001, r = -0.41), proportion-word (z = -4.84, p < .001, r = -0.48), proportion-notation (z = -5.22, p < .001, r = -0.52), and mixed formats (z = -6.03, p < .001, r = -0.60).

### Certainty and Comprehension

Significant positive correlations were found between self-ratings of certainty and comprehension of labels (rs = .414, df = 100, p =< .001), percentages (rs = .299, df = 100, p = .001) and pie charts (rs = .218, df = 100, p = .014). There were no significant relationships between certainty ratings and proportion-word (p = .137), proportion-notation (p = .286) and mixed formats (p = .302). The three formats that were easiest to comprehend showed a positive correlation between self-ratings of certainty and performance, whereas the formats that were less well understood showed no such relationship.

### Factors Affecting Probability Comprehension

A hierarchical, multiple regression was conducted to further examine the influences of age, verbal ability and gender on performance. This was conducted on total scores for the probability task ( = 12.63, SD. 3.79). Age was entered as the first determinant based on the results reported above. Age explained 26% of the variance in total score (F1,103 = 36.72, p =< .001) with BPVS scores explaining a further 7% of the variance (F2,102 = 25.46, p = .015). Thus, once age is controlled for higher verbal ability is associated with better understanding of probability. There was no effect of gender on total probability score.

## Discussion

The results of this study highlight two important messages for health professionals and researchers. Firstly, the results indicate that when information is provided in an appropriate format young children show evidence of understanding probability and are more confident about their judgements. This concords with previous research which has illustrated that in experimental conditions children can make probability judgements [61,38,40]. It therefore strengthens the argument for children to be included in communications of probabilistic health messages using developmentally appropriate materials.

Secondly, it was found that format significantly affects younger children's ability to understand probabilities. Health professionals should be aware that proportions are poorly understood and misunderstandings may trigger unwarranted concerns in children. For example, if children believe that 1 in 1000 is more likely than 1 in 10 utilising 1:1000 in an attempt to allay concerns is actually likely to make a child more anxious. This concords with previous work conducted with children which warns that they find this format problematic [40] and with research conducted with adults which demonstrates that misunderstandings arise from their use [61-63]. Despite this, research suggests that proportions are one of the most frequently used formats [26], specifically when conveying disease risk in genetic counselling sessions [64].

Research also shows that both genetic counsellors and parents use verbal labels, especially when conveying small probabilities [64,65]. Although children in both age groups performed well when using this format, the findings from this study warrant caution when using verbal labels as almost half of the participants requested an explanation. Concerns have also been raised regarding the use of verbal labels with adults [66] and adolescents [28]. Thus, health professionals should ensure that children understand the implications of such words when they are used within medical discussions. In addition, it has been shown that there can be ambiguity in how verbal labels are interpreted [11,51-55,67,68]. This suggests that designers of probabilistic health-related information should extensively research lay understanding of the magnitudes inferred by different verbal labels to ensure the correct message is being conveyed.

These results indicate that, of the formats studied, children are most likely to comprehend probabilistic information presented as either percentages or pie charts with 70% and 84% of children scoring maximally on these formats, respectively. The data suggest pie charts would be preferable as children performed best on this format and were also most confident about their responses, suggesting that this may be a good communication strategy with children. This finding is supported by evidence that graphical formats are optimal for conveying probability information to people with low literacy or numerical skills [29,30] and for retaining attention [30]. It is likely that these formats were easiest to understand as children can revert to using magnitude judgements to estimate probability. Magnitude judgements are simply the ability to judge which of two objects is largest. It has been argued that this skill precedes the understanding of probability [69] although even children with the capacity to reason using probabilities have been found to prefer to use magnitude judgements to solve probability tasks [70]. Although the use of percentages has been advocated with adolescents [28] it should be noted that this when performance was compared to verbal label comprehension. Additionally, research with adults suggests that whilst percentages are often used when discussing treatment options [66], they can be poorly understood [27,71].

The child's age was also found to affect performance on all formats with the exception of percentages. Although this is in line with previous research [37], the present study illustrates that these results should not be taken to indicate that younger children cannot understand probability. Rather it should be acknowledged that they may need more guidance to understand certain probability formats. No differences were found in performance between children who requested an explanation and those who did not require one, suggesting that with minimal guidance children can understand most new formats of probability. There is an important distinction to be made between explanations in relation to verbal labels and explanations for numerical representations of probabilities. Explanations for verbal labels are specific to a particular descriptor and cannot be easily generalized. Thus, explanations were commonly requested for each term in the verbal label format. The provision of explanations in numerical formats (e.g. percentages are how often something happens out of one hundred) could be generalized and therefore children were less likely to require an explanation in subsequent trials of that format. Thus, following the provision of guidance children may be able to make probability decisions using formats such as percentage and pie charts in the future, whilst children may consistently need support to understand the meaning conveyed by verbal labels.

An analysis of factors which contribute to overall probability understanding suggests that verbal IQ also contributes to performance once age has been accounted for, a finding supported by previous research [37]. Self-ratings of certainty showed a positive correlation with percentage, verbal labels, and pie charts. Comprehension, therefore, was linked with defendable estimates of certainty.

The verbal labels in the trials were selected as they are typically used to describe probability in health messages. Despite careful planning, consultation and piloting some children did not know a number of the words. This serves to emphasize that verbal labels may need extensive research to ensure the intended message is conveyed.

We acknowledge that we were not able to calculate response rates. This was not feasible within the ethical and school-based constrictions and it is relatively common that response rates are not reported in this field [37,72]. There is also a methodological reason why response rates can be potentially misleading in the reporting of school-based samples. Response rates are routinely used to gauge how representative a sample is, as a measure of how closely the sample recruited appropriates the population it was drawn from. As it is school teachers who select the children seen by researchers, a percentage figure may not be an adequate measure of how representative a given sample is. It is possible that teachers tend to select the most intelligent pupils (to present a good image of the school, or because these children have finished their work) or the children who are disruptive to the class. This bias may lead to an over-or underestimation of the abilities of children in general. A strength of this study was that the sample was drawn from a range of schools and was representative of the general population in terms of IQ.

The authors acknowledge that the measure of comprehension used in the cup game was somewhat limited as it was a measure of the ability to select which option was most likely. Clearly further detailed research is necessary to establish whether children can use certain formats more readily. Such studies could be based on modified experiments used with adults, such as asking them to select relevant information from materials to make judgements about treatment, or ascertain whether a test result is in the normal range [8]. The aim of the study, however, was to verify whether children aged 7–11 could make probability judgements and whether the format used would affect their ability to do so.

Further research utilising a modified version of this measure would be beneficial. It would be advantageous to include a wider range of graphical representations to establish whether the effects seen in this study were attributable to pie charts per se or graphical representations more generally. This recognises the mixed findings regarding the utility of pie charts [22,73-76]. Also, extending the number of trials of each format would enable one to explore more fully the range and extent of children's ability to make judgements utilising these formats and a clearer distinctions between which formats represent optimal communication strategies.

Finally, the results of this work needs to be verified in applied settings. While it can be concluded that children can understand certain probability formats when presented as classroom exercises, it cannot be assumed that children will readily adapt this knowledge to a clinical setting where the probabilistic message has personal implications.

## Conclusion

In conclusion these results demonstrate highly significant effects of format on comprehension. A more in-depth exploration indicated that performance across formats was more variable in the younger age group. Asking children of all ages to make judgements across different formats significantly reduced their ability to select the highest probability correctly. Although children performed well when presented with verbal labels, percentage and pie chart formats, we conclude that pie charts were the optimal format to enable children to make decisions about probabilities within this study. We have concluded that pie charts are optimal because, although children performed well on other formats such as percentages and verbal labels, pie charts consistently showed the best levels of performance. This was true regardless of whether one examined mean scores, percentage of children gaining maximum points or median and interquartile ranges. Additionally, children reported that they were most certain of their answers using this format.

These finding have clear implications for health professionals' communication and the involvement of children in health care. Health professionals should be encouraged that our results suggest that children may be able to understand simple risk information and that the use of an appropriate format can enhance their understanding. Although the focus of this study was to establish the best format in which to convey probabilistic information regarding health to children with a specific focus on genetics, the results of this study could clearly also be used in other health and non-health related areas.

## Competing interests

The authors declare that they have no competing interests.

## Authors' contributions

FU conceived of the study, designed the study, co-ordinated and carried out the data collection and analysis, and drafted the manuscript. CG & ET participated in the design of the study, data analysis and redrafting of the manuscript. All authors read and approved the final version of the manuscript.

## Pre-publication history

The pre-publication history for this paper can be accessed here:

http://www.biomedcentral.com/1472-6947/9/26/prepub
